# One-year results of drug-coated balloons for long and occlusive Femoropopliteal artery disease: a single-arm trial

**DOI:** 10.1186/s12872-020-01356-w

**Published:** 2020-02-06

**Authors:** Zhichao Lai, Xin Zhang, Jiang Shao, Kang Li, Lijing Fang, Leyin Xu, Xiaoxi Yu, Jingjing Wang, Xiu Liu, Jinsong Lei, Bao Liu

**Affiliations:** Department of Vascular Surgery, Peking Union Medical College Hospital, Peking Union Medical College and Chinese Academy of Medical Sciences, Beijing, China

**Keywords:** Drug-coated balloon, Femoropopliteal artery, Endovascular intervention

## Abstract

**Background:**

The performance of drug-coated balloons (DCBs) in femoropopliteal interventions has been proven through randomized trials in short lesions and lesions with relatively low proportion of occlusions. There is limited evidence of DCBs in long or occlusive lesions. This study is to investigate the efficacy of the paclitaxel-coated balloon for treatment of long and occlusive femoropopliteal arterial lesions.

**Methods:**

A single-arm trial including 44 femoropopliteal lesions (chronic total occlusion (CTO) plus > 10 cm) treated with DCBs was performed to collect data of average 1-year follow-up. Endpoints contain primary patency, target lesion revascularization (TLR), amelioration of the Rutherford classification, change of ankle brachial index (ABI) and major adverse events.

**Results:**

Technical success is 97.7% while device success is 100%. Mean lesion length was 186 ± 86.3 cm. Stent implantation was performed in 13.6%. Cumulative probability of primary patency was 78.8% ± 6.8% at 1 year while that of freedom from TLR was 91.4% ± 4.9%. Rutherford classification improved from average 3.3 ± 1.0 to 2.1 ± 1.4 (*p* < 0.001) at follow-up with a 72.7% amelioration rate. Ankle-branchial index changed from 0.33 ± 0.40 to 0.67 ± 0.37 (*p* = 0.002). No major adverse event was observed.

**Conclusion:**

These results suggest that it is safe and effective to treat long and totally occlusive femoropopliteal artery disease with DCBs. Further studies are demanded to confirm these results.

## Background

“Leave nothing behind” strategies are admitted by an increasing number of surgeons. Compared with stents, deployments of balloons can leave a better condition for future possible repeated angioplasty. However, plain old balloon angioplasty seemingly performs not well, especially when used in long and complex lesions [1, 2].

To have a better therapeutic effect, drug-coated balloons (DCBs) are an attractive alternative which can inhibit proliferation of tunica intima and restenosis. Many studies of DCBs have proved the superiority on the efficacy and safety in arteriosclerosis obliterans of low extremities, although recently there are some controversies of long-term safety. But most studies focus on the usage of DCBs in relatively short lesions in femoropopliteal arteries and have a relatively low proportion of chronic total occlusion (CTO) lesions [3–7]. The data of prognosis about long and occlusive lesions is very limited and insufficient [1, 2, 8]. Among published article, the result of CTO cohort in IN.PACT Global study by Gunnar Tepe et al. is the only long CTO DCB outcomings, while there are several CTO studies about self-expandable stents [9]. However, long and occlusive lesions are very common challenges in clinical work and tend to have worse outcomings than shorter lesions. It is necessary to have more results about treatment of long and totally occlusive arteriosclerotic lesions with DCBs for future clinical reference.

With this objective of providing evidence of DCBs in long and occlusive lesions in femoropopliteal arteries, we herein report 1-year follow-up data of patients with CTO femoropopliteal lesions longer than 10 cm. Most past studies with long lesions have proportion of CTO ranging from 40 to 70% [1, 2, 10]. Although there have been IN.PACT CTO study result reported [9]. It is still the first time to report data from Asian population of long lesions with 100% proportion of total occlusion.

## Patients and methods

This study was an independent, single-center and single-arm trial of patients with femoropopliteal long and occlusive lesions (excluded stenosis-only lesions) and treated with DCBs, whose objective was to appraise their outcomes in details. The study was retrospectively performed with the DCB data pool of our center. (All patients treated by DCBs were included in this pool and have regular follow-up data. The intervention procedures related to this pool performed by the five operators at our center were standardized before.) All patients have signed informed consent before the procedure. This study was approved by the local institutional review board.

### Patients

Procedures were performed from July, 2016 to March, 2018. Adult patients undergoing treatment of long and occlusive femoropopliteal atherosclerotic lesions with DCBs were screened. Rutherford stages range from 1 to 5 (claudication, rest pain or small ulcer). Angiographic inclusion criteria include lesions in superficial femoral and/or popliteal artery, with minimum lumen diameter being 0 and a total length ≥ 100 mm. Multiple adjacent lesions without angiographic evidence of healthy segments 3 cm or larger were considered and treated as single lesions. All patients included have at least 1 patent outflow vessel (< 50% diameter stenosis) before treatment of the femoropopliteal lesions. If there is any lesion in inflow vessels able to be successfully treated before the target femoropopliteal lesion, the patients were deemed eligible. In exclusion criteria, patients who is nonatherosclerotic disease such as aneurysm and vasculitis and patients with any alternative therapies, including atherectomy, excimer laser or cutting balloon during the index procedure, were excluded. Patients who did not adhere to post-procedure dual antiplatelet therapy over 1 month were also excluded.

The capture of follow-up information was at 3 months, 6 months, 1 year and 2 years after the intervention. The mean follow-up time of patients in this study was 388 ± 217 days after procedures.

### Procedures and devices

Before procedures, every patient took aspirin 100 mg. QD and clopidogrel 75 mg. QD for at least 7 days. If patients did not have recent medical history of aspirin or clopidogrel, they would take both for 300 mg 12 h before operations. After procedures, patients had to take 100 mg/day aspirin and 75 mg/day clopidogrel for a minimum of 12 weeks. Most patients are recommended to take atorvastatin 20 mg qd after procedure, no matter how the baselines of low-density lipoprotein cholesterol (LDL-C) are. Besides, if patients take statins before procedure due to hyperlipidemia, it will continue after the procedure.

During the procedures, 75 IU/kg heparin were administered in each patient after sheath insertion. Most occlusions were passed through intraluminal method while subintimal method was also applied when necessary according to operators’ discretion. Pre-treatments before DCBs were pre-dilation (2 min) with uncoated balloons (0.5 to 1 mm smaller than reference vessel; visually estimated). DCBs were 1.0 mm larger than uncoated balloons and inflated only once for 3 min at 6 to 12 atm If two DCBs were used for only one lesion, there were at least 5 mm overlaps between the dilations, since there are 400 mm lesions and the longest Orchid balloons we used are 300 mm.

If any persistent stenosis (> 30%) or flow-limiting dissection existed, additional dilations of uncoated balloon would be considered. After repeat dilations, if persistent stenosis or flow-limiting dissection still existed, bailout stents would be deployed.

DCBs used in our center are all Orchid drug-coated balloon by AcoTec company with diameters 4-6 mm and length 120-300 mm. Provisional stents include Zilver Flex, Protégé Everflex and EverCross stents. All procedures above can be adjusted to some extent under operator’s discretion. All operators are chief or associate chief surgeons with sufficient intervention experience. The retrograde access was used twice in 44 patients while subintimal passage method was used once.

### Definition and study endpoints

Device success is defined as the successful access and exact deployment of the device to the target lesion, while technical success is defined as meeting the requirement of device success and the residual stenosis < 30% in visual estimation after endovascular procedure.

The primary endpoints of this study include primary patency, freedom from clinically driven target lesion revascularization (TLR) and major adverse events (including death of any cause and major target limb amputation). Primary patency is defined as no restenosis proven through clinically driven TLR or evidence of < 50% residual lumen diameters in the target lesion under computed tomography angiography and peak systolic velocity ratio > 2.4 under doppler ultrasound examination. Clinically driven TLR is defined as any reintervention of the target lesion because of apparent symptoms or imaging results. Secondary endpoints are the change of Rutherford stage (degrees of claudication were classified by meters patients can walk until appearance of symptoms of claudication. Stage 2 ranges 300–500 m before appearance of claudication.) and the change of ankle brachial index (ABI). Calcification was evaluated as PACSS scale [11, 12]. Sever degree was defined as two sides of vascular walls have calcification and one of them was longer than 5 cm.

### Statistical analysis

The data were analyzed on the per-protocol population. Descriptive statistics were used to estimate values and changes from baseline as absolute frequencies and percentages for categorical variables and mean ± standard deviation and medians for continuous variables.

Primary patency and freedom from TLR were estimated by the Kaplan-Meier analyses. Comparison of Rutherford classification and ABI between pre-procedure and follow-up was calculated through Wilcoxon rank sum test. Cox proportional hazards multivariate regression analysis was used to estimate the influence of potential prognostic factors: sex, lesion length (< 150 mm vs. ≥150 mm vs. ≥250 mm) smoking, hypertension, diabetes and calcification. Statistical significance was considered when *p* values < 0.05.

## Results

### Baseline and procedural characteristics

Between August 2016 and March 2018, totally 44 patients with 44 femoropopliteal lesions were eligible for this study treated with 48 DCBs. The mean age was 68 ± 12 years old and 75.0% of the population was male. The majority of population had hypertension (70%) and smoking (57%) with Rutherford classification 3 or 4 (43 and 30%, respectively). And half of patients had diabetes. (Table 1).

**Table 1:** Baseline Clinical Characteristics (*N* = 44)

Age (years)	68 ± 12
Male	33 (75)
Hypertension	31 (70)
Hyperlipidemia	10 (22)
Diabetes	16 (50)
Prior/Current Smoking	22 (57)
Renal failure	1 (2)
Coronary arterial diseases	12 (27)
Cerebrovascular history	11 (25)
Rutherford Class	
1	3 (7)
2	4 (9)
3	19 (43)
4	13 (30)
5	5 (11)
6	0 (0)
Lesions number	44
Lesion type	
De novo	33 (75)
Restenosis	11 (25)
Calcification	
None or slight	39 (89)
Moderate or Severe	5 (11)
Lesion length(mm)	186 ± 86.3
Stenosis degree	
Slight or Moderate	0
Severe	0
Total occlusion	44 (100)
TASC II	
B	19 (43)
C	6 (14)
D	19 (43)
BTK outflow	
3 vessels	26 (59)
2 vessels	5 (11)
1 vessel	12 (27)
None	1 (2)

^a^Values are % (n) or mean ± SD

The mean lesion length was 186 ± 86.3 mm with TASC II classification B to D (43, 14 and 43%, respectively) and 75% de novo lesions. All included were total occlusion while only 11% is regarded as moderate or severe calcification (defined as compromising both sides of the arterial wall for at least 5 cm). (Table 1).

DCB/lesion ratio, which is defined as the mean number of DCBs used per lesion, was 1.091 and DCBs were with diameter of 4.59 ± 0.68 mm and length of 196.9 ± 76.9 mm which is 10 mm longer than the mean lesion length. 6 bailout stents were used in 44 lesions (13.6%). The retrograde access was used twice in 44 patients while subintimal passage method was used once. (Table 2).

**Table 2:** Procedural characteristics (*N* = 44)

Lesions	44
Technical Success	97.7% (43/44)
Device Success	100%
DCB/lesion ratio	1.091
Mean DCB diameter (mm)	4.59 ± 0.68
Mean DCB length (mm)	196.9 ± 76.9
Stenting	13.6% (6/44)
Retrograde Access	4.54% (2/44)
Subintimal Passage Method	2.27% (1/44)

^a^Values are % (n) or mean ± SD

After procedure, 34 of 44 patients (77.3%) took atorvastatin 20 mg qd to further decrease LDL-C and restenosis rate.

### Efficacy and safety outcomes

**Fig. 1:**
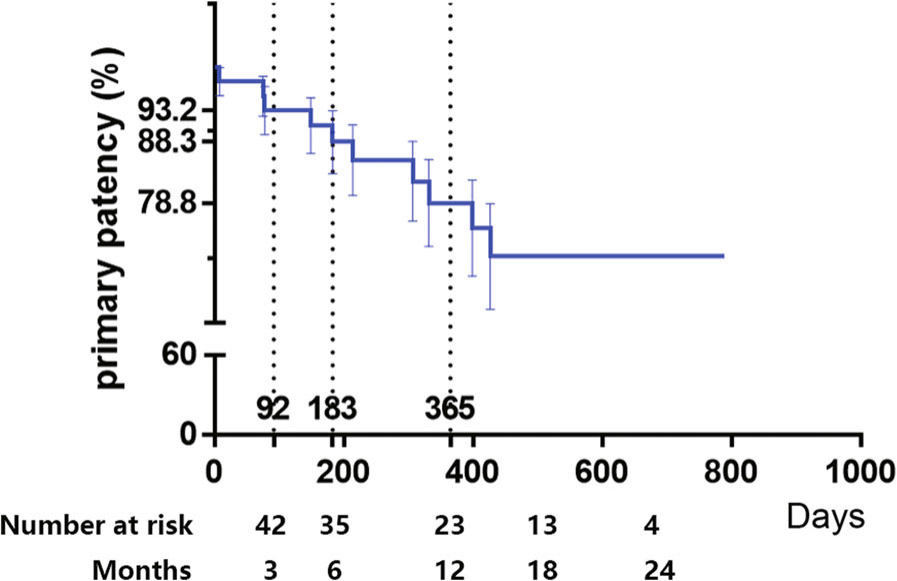
Primary Patency Endpoint. Kaplan-Meier curves shows cumulative probability of primary patency of this study over an average 1-year follow-up period

**Fig. 2:**
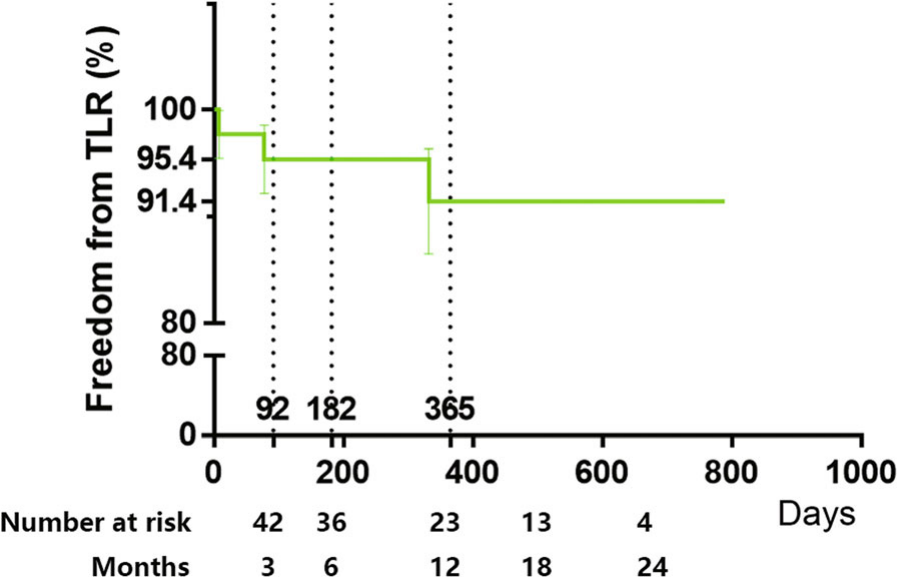
Freedom from TLR Endpoint. Kaplan-Meier curves shows cumulative probability of freedom from TLR of this study over an average 1-year follow-up period

Device success is 100% while technical success is 97.7%. Cumulative probability of patency by Kaplan-Meier estimate was 93.2% ± 3.8% at 3 months, 88.3% ± 4.9% at 6 months, 78.8 ± 6.8% at 1 year and 70.4% ± 8.3% at 2 years (Fig. 1). The rate of freedom from TLR was 91.4 ± 4.9% after 1 to 2 years (Fig. 2).

**Fig. 3:**
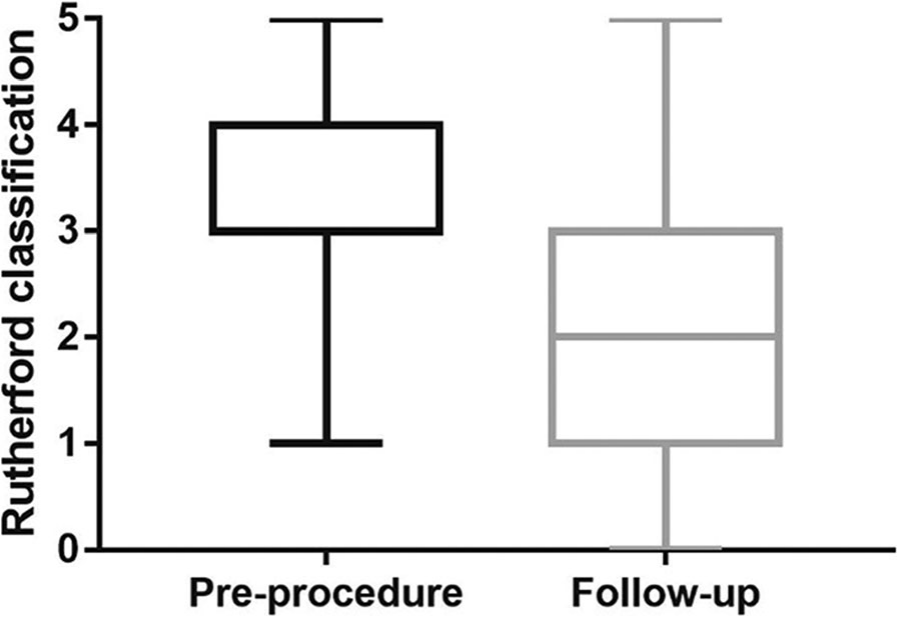
Functional Results (Rutherford classification). Rutherford classification of pre-procedure and 1-year follow-up was compared

The mean Rutherford classification of pre-intervention versus follow-up was 3.3 ± 1.0 versus 2.1 ± 1.4. The amelioration is statistically significant (*p* < 0.001). The amelioration of Rutherford classification was displayed in Fig. 3. Clinical improvement of at least 1 Rutherford classification between pre-intervention and follow-up was in 72.7% of limbs, while no change in 18.2% and deterioration in 9.1%. The ABI changed from 0.33 ± 0.40 (before procedures) to 0.67 ± 0.37 (at 1-year follow-up), statistically significant (*p* = 0.002). The ABI values after procedures but still hospitalized are not included in this study.

No statistically significant association with the primary endpoint was found in the Cox proportional hazards model for sex, smoking, lesion length, TASC II stages, hypertension or diabetes.

As for safety outcomes, there was no procedure- or device-related death or major adverse event through average 1 year.

Main outcomes of this study were listed in Table 3.

**Table 3:** Key Outcomes

Outcome	n (%)	95% CI	Cumulative Probability
Primary Patency	34 (77.3)	64.2–89.2%	78.8%
Freedom from TLR	41 (93.2)	85.4–100%	91.4%
Amelioration of Rutherford Classification	32 (72.7)	59.0–86.4%	/
	Pre-procedures	Follow-up	*P* Value
Rutherford classification	3.3 ± 1.0	2.1 ± 1.4	< 0.001
Ankle-Branchial Index	0.33 ± 0.40	0.67 ± 0.37	0.002

No independent predictors or major safety endpoints were found

## Discussion

In recent years, the studies of DCBs treating atherosclerotic lesions in femoropopliteal arteries have proven favorable results of patency and freedom of TLR. But these studies mainly focused on lesions relatively shorter and stenotic rather than completely occlusive. Many randomized controlled trials (RCTs) have reported results over 3 to 5-year outcomes with shorter lesions. 1-year TLR rate is from 2.4% to 12.3% with mean lesion lengths ranging from 60 mm to 90 mm. 1-year primary patency is from 75.8% to 87.3%. Even 5-year primary patency rate (THUNDER trial) is over 70% [10, 13, 14]. Nevertheless, long and occlusive lesions are very common and can result in higher possibility of subintimal passage and losing more dose of paclitaxel in passage [15]. For example, if subintimal passage method is used, paclitaxel will contact more with smooth muscle cells other than endothelial cells, which is still controversial and may bring potential differences. (It has been proven in some studies that even smooth muscle cells which do not directly contact the paclitaxel coating can be inhibited effectively in proliferation and migration in vivo [16]. There are also evidences that short-term application of paclitaxel is more significant on smooth cells than on endothelial progenitor cells [17]. However, it is reported no statistical significance found in comparison of intraluminal and subintimal group in long lesions [2].)

Thus, the results of long and occlusive lesions are very important and needed in clinical work. Until our submission, there are limited articles about long lesions with DCBs. Only the IN.PACT CTO study published in 2019 is focused on long and occlusive lesions and the DCBs [9]. But this is the first study including long and CTO femoropopliteal lesions totally based on Asian population.

In our results, the Kaplan-Meier estimate results of patency rate at average 1-year follow-up is 78.8% ± 6.8%, which is as expected. The IN.PACT CTO study with also 100% CTO lesions and 22.83 cm lesion length showed the primary patency is 88.7% (*n* = 126) [9]. In comparison with other long lesion study, Micari et al. reported 1-year primary patency in femoropopliteal lesions over 150 mm (*n* = 101) to be 83.2% while 1-year primary patency in complex femoropopliteal lesions (lesions over 10 cm or restenosis) was reported by Schmidt et al. as 79.2% [2, 8]. In comparison, 1-year patency rates of shorter lesions with DCBs range mainly from 85 to 90% (although some results are within 75–85%) which is higher than that of long-lesion studies to some extent [18–20]. This is probably because long lesions tend to cause higher possibility of subintimal passage and losing more dose of paclitaxel in passage. But since the difference is only 5–10%, the availability of paclitaxel in longer lesions is also satisfactory [15]. There are several studies of self-expandable nitinol stents treating with CTO lesions. The one-year results of primary patency rate are about 72 to 80%. But whether DCBs or traditional devices are better in long and CTO lesions needs further studies [21, 22].

The provisional stent rate in this study is about 13.6% while that in IN.PACT CTO study is 40.7% [9]. For further reference, most long but non-CTO study has provisional stent rates ranging from 10.5 to 23.3% but it could be highest to be 36.6% for severely calcified lesions [2]. It is possible that operators in our center will try their best and repeat tries to use intra-luminal method other than subintimal which has lower risk of dissection and residual stenosis. In this study, subintimal method is only used once in 44 patients. The other possible reason is that some operators prefer to pre-dilate to have larger luminal diameters while some operators tend to pre-dilate less to decrease the rate of dissection.

Meanwhile, both Fig. 1 and Fig. 2 show that there is almost no restenosis or TLR beyond 450 days after procedures. In some other long-term studies, this shape of curves also exists. For example, in the 2-year long lesion results of Micari et al., the most obvious decrease of primary patency is also between 300 and 400 days after procedures from about 95 to 75%, and the curves after 400 days are also relatively parallel [1]. Also, in the 3-year results from Schneider et al., decrease of primary patency is mainly two periods, 0–15 months and 24–27 months [10]. However, some studies, such as 2-year complex lesion results by Schmidt et al., show patency rates decreased in a relatively constant speed [2]. It is possible that potential unknown risk factors are more likely to cause restenosis within 450 days and patients without these factors can keep patent over 450 days.

The changes of the Rutherford classification and ABI are favorable, both statistically significant and consistent with expectation. Mean ABI changed from 0.33 to 0.67 and Mean Rutherford classification changed from 3.3 to 2.1 with 72.7% amelioration rates. In comparison of several similar studies, ABI changes from 0.49–0.63 to 0.82–0.95 while ABI changed from 2.4–3.4 to 0.5–2.0 [2, 8, 23].

The estimate of potential prognostic factors here failed to find any one statistically significant, probably due to the relatively small sample size. But in studies with complex lesions and large sample size, sex, obesity and severe calcification are regard as independent predictors [2]. In those studies, higher restenosis risk of female may relate to smaller lumen diameters while severe calcification means higher risk of recoil and residual stenosis and also interference with the transfer and deposition of the paclitaxel [24, 25].

Fortunately, no death or major amputation was found in this study. In most studies of DCBs, there is also no obvious adverse event of DCBs found. Since the only difference between DCBs and plain old balloons is paclitaxel which can decrease thickness of arterial wall, it is theoretically possible to cause ectasia and aneurysm. According to existing literatures, a limited number of aneurysmal dilations after drug-eluting stents or DCBs are only reported in coronary arteries, tibial arteries and femoral-to-below knee popliteal bypass grafts [26, 27]. However, in recent 1 year, there has been concern about all-cause death of DCBs. It is hitherto controversial and the Food and Drug Administration of the U.S. recommended to change from DCBs to alternative methods. Thus, more data and further studies about long-term safety are needed to confirm this suspicion.

### Limitations

This study is not randomized or controlled trial and is single-center, which may cause obvious bias. There are patients not attending scheduled follow-up and temporarily lost. Not all doppler ultrasound examinations were performed in our center, which means there is assurance of accuracy. And dropout can be seen to some extent.

## Conclusions

This study focused on long and totally occlusive femoropopliteal arterial atherosclerotic treatment with DCBs. Most lesions (78.8) with DCBs at 1-year follow-up can keep patent, although there were a small number of bailout stents used. Loss of patency mostly happened before the post-operative 450th day. TLR only performed in 6.8% patients which is much less than the proportion of restenosis. Ameliorations are seen on the Rutherford classifications and ABI, both statistically significant. And no major safety endpoint was observed.

## Data Availability

The datasets generated and analyzed during the current study are available from the corresponding author on reasonable request.

## Abbreviations

ABI: Ankle brachial index
CTO: Chronic total occlusion
DCB: Drug-coated balloon
LDL-C: Low-density lipoprotein cholesterol
TLR: Target lesion revascularization
